# Woody and Foliage Biomass, Foliage Traits and Growth Efficiency in Young Trees of Four Broadleaved Tree Species in a Temperate Forest

**DOI:** 10.3390/plants10102155

**Published:** 2021-10-11

**Authors:** Bohdan Konôpka, Jozef Pajtík, Vladimír Šebeň, Peter Surový, Katarína Merganičová

**Affiliations:** 1 National Forest Centre, Forest Research Institute, T. G. Masaryka 22, 960 01 Zvolen, Slovakia; bohdan.konopka@nlcsk.org (B.K.); jozef.pajtik@nlcsk.org (J.P.); 2 Faculty of Forestry and Wood Sciences, Czech University of Life Sciences Prague, Kamýcká 129, 165 000 Prague, Czech Republic; surovy@fld.czu.cz (P.S.); katarina.merganicova@forim.sk (K.M.); 3 Department of Biodiversity of Ecosystems and Landscape, Institute of Landscape Ecology, Slovak Academy of Sciences, 949 01 Nitra, Slovakia

**Keywords:** common aspen, European hornbeam, silver birch, sycamore, leaf weight and area, foliage and woody parts biomass

## Abstract

The main goal of this study is to analyse and interpret interspecific differences in foliage biomass/area and woody parts biomass as well as the ratio between quantities of foliage and woody components (i.e., branches, stem and roots). The study was principally aimed at determining basic biomass allocation patterns and growth efficiency (GE) of four broadleaved species, specifically common aspen (*Populus tremula* L.), European hornbeam (*Carpinus betulus* L.), silver birch (*Betula pendula* Roth.) and sycamore (*Acer pseudoplatanus* L.) in young growth stages. We performed whole-tree sampling at 32 sites located in central and northern parts of Slovakia. We sampled over 700 trees and nearly 4900 leaves to quantify biomass of woody parts and foliage traits at leaf and tree levels. Moreover, we estimated specific leaf area in three parts of the crown, i.e., the upper, middle and lower thirds. We found that hornbeam had the largest foliage biomass and the lowest foliage area of all investigated species, while its biomass of woody parts did not differ from aspen and sycamore. Birch had the lowest biomass of woody parts, although its foliage properties were similar to those of aspen. Intraspecific differences of foliage were related to tree size and to leaf position along the vertical crown profile. Growth efficiency (GE), expressed as woody biomass production per foliage area unit, was evidently larger in hornbeam than in the other three broadleaves. We suggest that future GE modelling should utilize real values of stem diameter increment measured in a current year, bio–sociological position of trees and competition indicators as inputs. Such an approach would elucidate the role of stand structure and tree species mixture for ecological and production properties of forest stands.

## 1. Introduction

Scientific interest in estimating tree biomass and its structure have been mostly focused on two principal research areas, specifically tree physiology and forest ecology [1]. Since tree biomass is the exclusive result of photosynthesis, which takes place in foliage, it is essential to understand tree growth considering at least two basic biomass components: woody parts (branches, stem and roots) and leaves. This kind of studies might elucidate growth strategies with regards to optimizing biomass allocation under certain ecological conditions [2]. Growth of particular tree components (i.e., biomass allocation) is ruled by a variety of internal (e.g., genetic properties, health status [3]) and external factors, especially climate, soil and stand conditions [4]. For instance, Konôpka et al. [5] showed that while the contribution of woody parts to total biomass of four tree species in Slovakia increased with the increasing tree size, the contribution of foliage diminished. However, genetic variability is a substantial factor in biomass allocation patterns resulting in interspecific differences [6].

Besides fulfilling physiological roles, foliage stores carbohydrates and mineral nutrients [7]. Moreover, foliage is a relatively short-living tree organ, and, in addition to photosynthetic and respiratory functions, it can substantially contribute to biochemical cycling in a forest ecosystem including carbon fluxes [8,9]. On the other hand, woody components remain active during the nearly whole tree life and thus sequester carbon for a long period [10].

As for leaves, some studies (e.g., [11,12]) showed significant modifications of their size and mass density, mostly in response to contrasting light conditions. Light conditions in a forest stand are often related to a position of a tree in the canopy as well as to a vertical position along the tree crown [13]. The most frequent indicator of foliage structure is specific leaf area (SLA; [14]). In principle, a leaf has a lower SLA if it has a greater mass per volume or if it is thicker. Besides the morphological background of SLA, the indicator can be implemented as a conversion factor for calculating foliage weight (measured variable) to area (modelled variable). The leaf mass ratio (LMR), the ratio of dry leaf mass to total dry plant mass, as well as the leaf area ratio (LAR), the ratio of leaf area to total dry plant mass, have been previously used to describe the interaction between the ecological structure and tree production (e.g., [15,16]).

In general, research that considers the biomass of both woody parts and foliage traits (e.g., foliage weight and area, SLA) is rare, although the ratio between the mass increment of woody components in trees and their foliage mass or foliage area can help identifying the growth efficiency of assimilatory organs of tree individuals (or tree species).

The relationship between foliage and biomass growth is strongly related to productivity. Growth efficiency (GE; see [17]) can be expressed as stem biomass (alternatively, biomass of woody parts) increment per foliage biomass unit or per foliage area. Interspecific comparisons of GE could help us to elucidate ecological demands and growth strategies of multiple tree species [18]. Different foliage growth and retention strategies of species in relation to light availability might be important for carbon stock and cycling in forest ecosystems and should be considered in forest management [19]. Such knowledge might be implemented in forest management planning, for example in decision-making about suitable tree species composition for a particular site beneficial for multiple forest functions, biodiversity, carbon sequestration, climate change mitigation and adaptation [20].

While we still have a large proportion of global area covered by relatively old or mature stands, over recent decades, the area of regenerating and young forests has been significantly expanding due to the damage inflicted on forests by disturbances (windstorms, drought episodes, bark beetles and forest fires; e.g., [21]). Moreover, the frequency and intensity of these disturbances are increasing as a result of climate change. The difference in biomass production and allocation between young and mature trees is often quite stark. Thus, existing and rather frequent models for mature trees are not applicable to young ones (e.g., [22]). From the initial stand establishment, through competition growth and into maturity, the foliage quantity relative to other biomass compartments change [23]. To account for these changes, we urgently need to fill the gap in data sets and models focusing on small trees, their traits and biomass, and its partitioning. Therefore, the primary goal of this study was to quantify and discuss interspecific differences in foliage area, foliage biomass, and woody parts biomass as well as the ratio between quantities of foliage and woody components. We studied four broadleaved species, specifically common aspen (*Populus tremula* L.), European hornbeam (*Carpinus betulus* L.), silver birch (*Betula pendula Roth.*), and sycamore (*Acer pseudoplatanus* L.). The chosen species do not usually form pure stands but are part of mixed species stands in temperate European forests. Nevertheless, their importance may increase in future due to changing environmental conditions, which may disfavour the majority of current economic tree species (e.g., [24]). These represent species with rather different morphological functional properties of leaves, woody parts and ecological demands (see e.g., [25,26]). Alongside presenting models for individual tree species, this work also contributes to interspecific comparisons of biomass allocation, foliage traits and GE. Since the selected tree species represent genera with contrasting ecological demands (e.g., common aspen as light demanding versus European hornbeam as shade-tolerant), we also expected interspecific differences in the foliage traits, biomass and GE.

## 2. Results

**Figure 1:**
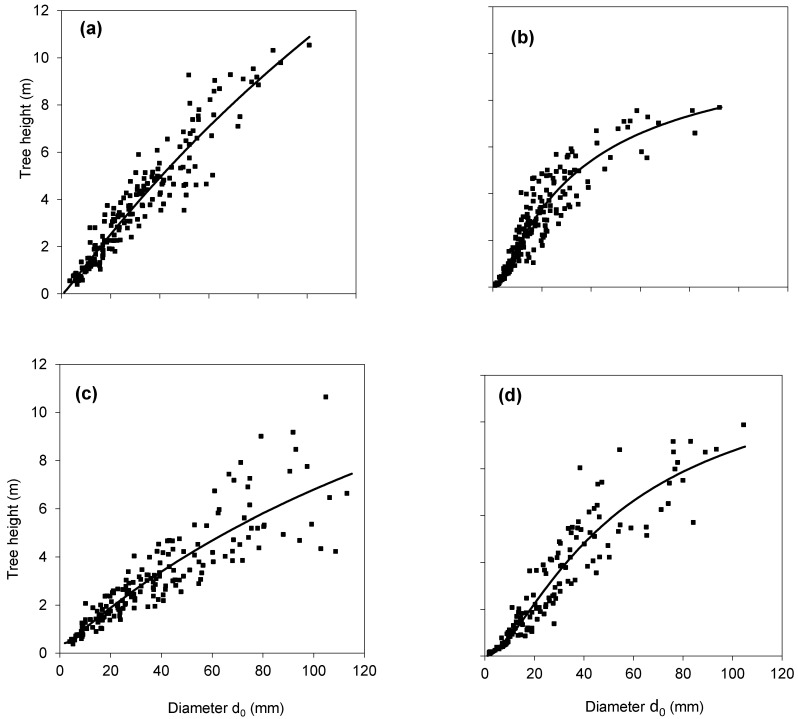
Relationships between diameter at stem base *d*_0_ and tree height for four broadleaved species, specifically common aspen (*Populus tremula* L.) (**a**), European hornbeam (*Carpinus betulus* L.) (**b**), silver birch (*Betula pendula Roth.*) (**c**) and sycamore (*Acer pseudoplatanus* L.) (**d**). Equation: $h=\frac{d_{0}^{2}}{b_{0}+b_{1}+b_{2}d_{0}^{2}}$, its parameters and statistical characteristics are given in Table 1.

Relationships between diameter *d*_0_ and tree height indicates interspecific differences in tree height for individuals of certain diameter (Figure 1). If we consider the same value of *d*_0_, trees of the common aspen were the tallest, followed by sycamore and hornbeam, and the smallest were silver birch trees. For instance, modelled tree heights of trees with *d*_0_ diameter equalling 60 mm were (according to fitted curves describing relationships between *d*_0_ diameter and tree height) 7.3 m, 6.8 m, 6.7 m and 4.9 m for aspen, hornbeam, sycamore and birch, respectively.

**Figure 2:**
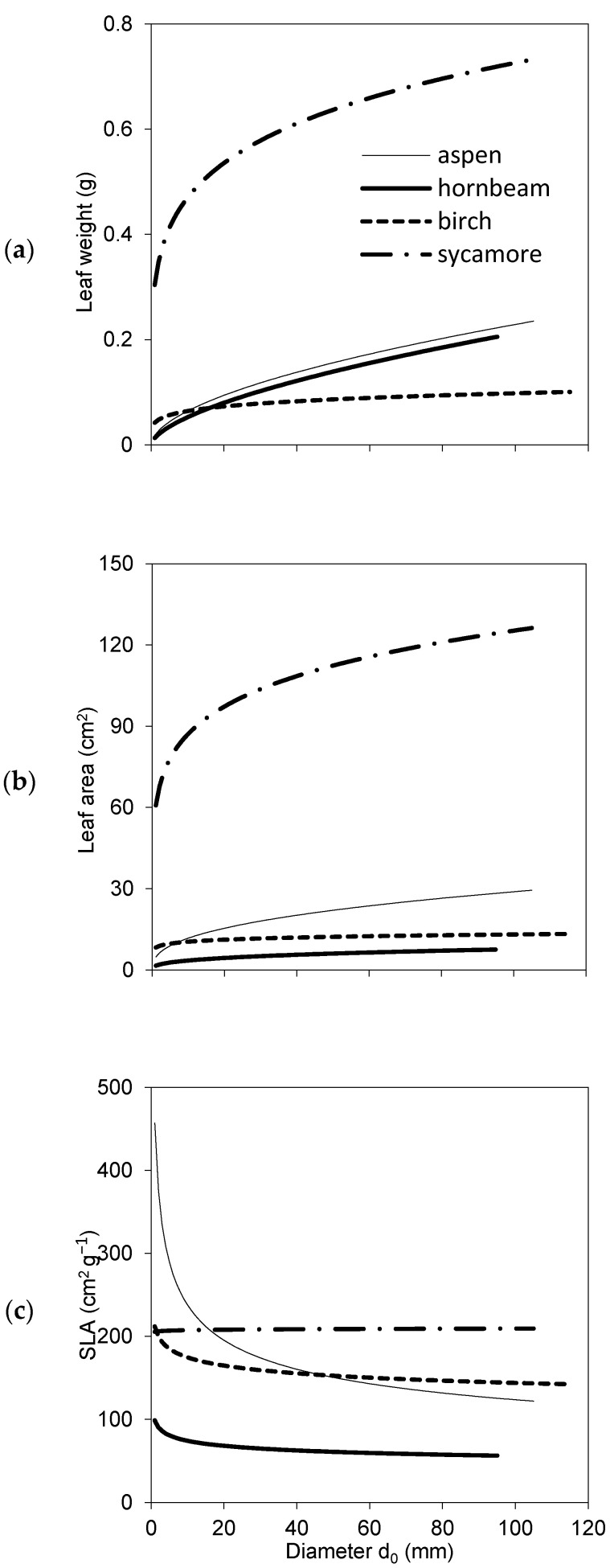
Species-specific models for individual leaf weight (**a**), leaf area (**b**) and specific leaf area (SLA) (**c**) expressed against diameter *d*_0_ (see Table 2 for parameters and statistical characteristics of equations).

Our measurements showed both intraspecific and interspecific differences in leaf characteristics, i.e., weight, area and SLA (Table 2, Figure 2). If we consider a tree crown as a whole, the heaviest individual leaves were recorded for sycamore, followed by common aspen and European hornbeam, while silver birch had the lightest individual leaves from all investigated species. As for leaf area, the order was slightly different (shown from largest to smallest): sycamore, common aspen, silver birch and European hornbeam. The largest values of SLA of individual leaves were found for sycamore, slightly smaller for the common aspen and silver birch, and much smaller ones were recorded for the European hornbeam. The models demonstrated that both leaf weight and leaf area grew with diameter *d*_0_, while the greatest changes were observed in smallest trees (diameter *d*_0_ up to approximately 20 mm). The opposite trends were found for SLA, which decreased with the increasing diameter *d*_0_ for all investigated species except for sycamore, which had a constant SLA regardless of diameter.

**Figure A1:**
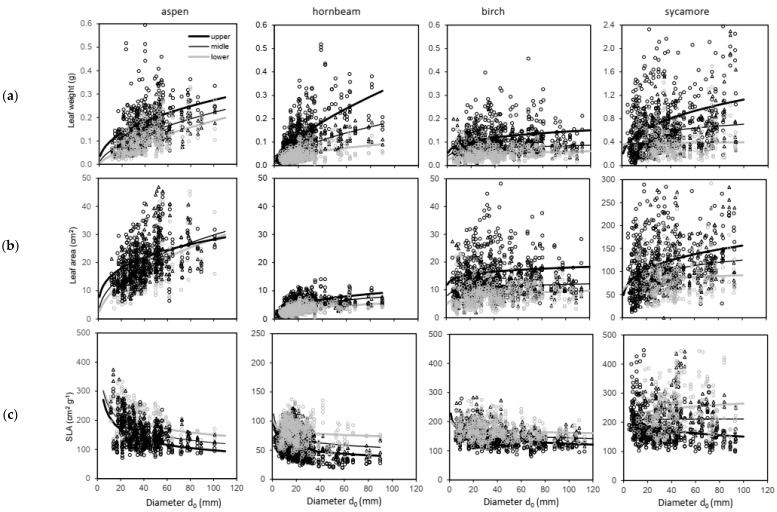
Species-specific models for individual leaf weight (**a**), leaf area (**b**), and specific leaf area (SLA, (**c**)) expressed for three parts of vertical crown profile, i.e., upper, middle and lower third against diameter *d*_0_.

We found that the variability of leaf characteristics was related to the position of leaves along the vertical crown profile (upper, middle and lower third; see Figure A1 and Table A1 in the Appendix A). The derived models for individual tree crown parts indicated differences between the foliage growing in their crown thirds. The heaviest leaves usually occurred in the upper crown part, while the lightest ones occurred in the lowest part regardless of the species. At the same time, the models revealed general trend of increasing leaf area and decreasing SLA from the bottom of the crown towards the treetop for all investigated species (check Figure A1 in the Appendix A). However, the variability in data was large, and a great proportion remained unexplained by the models derived for individual crown parts.

**Figure 3:**
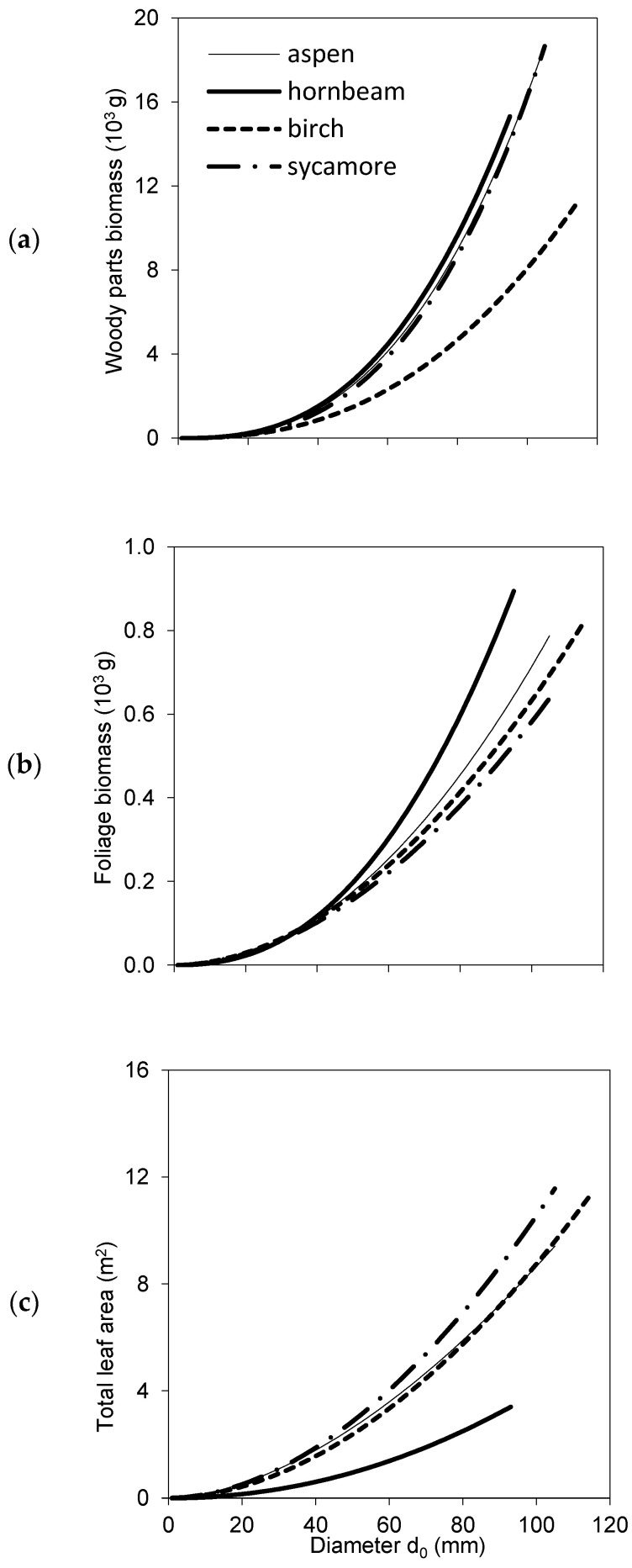
Species-specific models for woody parts biomass (**a**), foliage biomass (**b**) and leaf area at a tree level (**c**) against diameter *d*_0_ (see Table 3 and Table 4 for parameters and statistical characteristics of parameters).

Biomass models for woody parts and foliage (Table 3, Figure 3a,b) showed interspecific differences in the biomass of woody parts as well as of foliage. Birch trees had substantially less mass weight of woody parts in comparison to other species. From the point of view of foliage biomass, hornbeam differed from other species, as its leaves were markedly heavier. Hornbeam had also much smaller total tree foliage area than the other species (Table 4, Figure 3c), while sycamore had slightly larger foliage area than aspen or birch.

**Figure 4:**
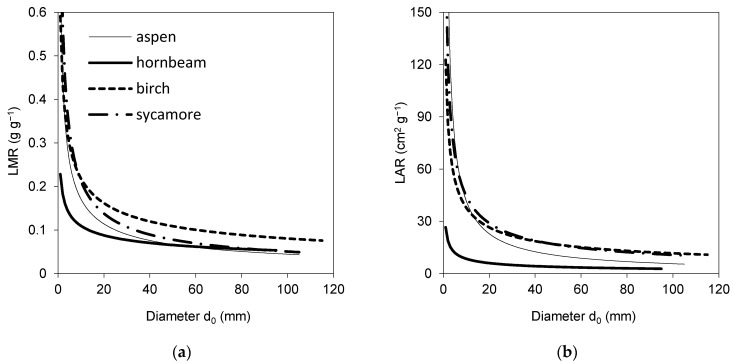
Species-specific models for leaf mass ratio (LMR) (**a**) and leaf area ratio (LAR) (**b**) against diameter *d*_0_ (see Table 5 for equation in Figure 1d parameters and statistical characteristics).

Data on foliage biomass and area and woody parts biomass at a tree level allowed us to derive models for LMR and LAR (Table 5, Figure 4). The models showed that both indicators obtained rather similar values for all tree species for a respective diameter *d*_0_, except for hornbeam, which manifested lower values of LAR than the other species.

**Figure 5:**
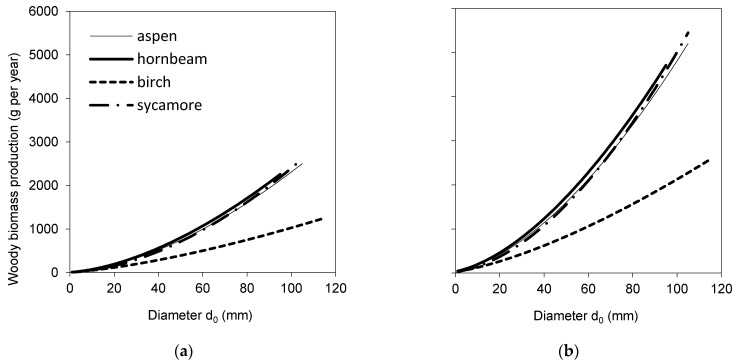
Species-specific models for the annual production of woody parts biomass, i.e., branches, stem and roots together, against the initial diameter *d*_0_ considering annual diameter increment of 5 mm (**a**) and 10 mm (**b**). Algorithms for calculation of the points on the curves are given in the Materials and Methods section (Equation (10)).

**Figure 6:**
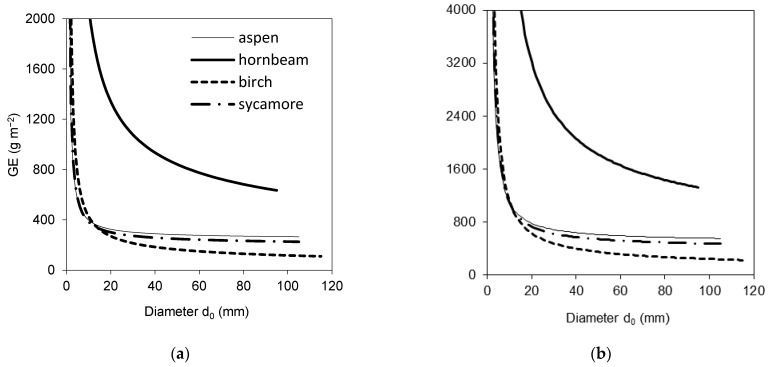
Species-specific growth efficiency (GE) presented against the initial diameter *d*_0_ considering annual diameter increment of 5 mm (**a**) and 10 mm (**b**). Algorithms for calculation of the points on the curves are described in the Materials and Methods section (Equation (11)).

Wood production of four broadleaved species was calculated for two potential annual stem base diameter increments, specifically 5 and 10 mm. The results indicated that, while hornbeam, birch and sycamore trees with the same initial diameter *d*_0_ had very similar wood production, birch produced substantially less woody biomass (Figure 5). The analysis of GE revealed that GE of hornbeam was much higher than that of the other species (Figure 6).

## 3. Discussion

The results suggested that the four studied broadleaved species had some similar characteristics, while other ones were rather contrasting. If we summarize these differences, we can conclude that common aspen trees were the tallest, and hence, they had the largest height to diameter ratio, while their other traits occurred in the middle of the value ranges revealed for all studied species together. European hornbeam differed from other species in many traits: its individual leaf area, SLA and total tree leaf area were the lowest (Figure 2 and Figure 3). In contrast, the total tree foliage biomass of hornbeam was the largest from all investigated tree species, while its biomass of woody parts was similar to that of aspen and sycamore. Consequently, hornbeam values of LMR and LAR were the lowest (Figure 4), and GE was the highest (Figure 6). Silver birch was found to have the lowest height to diameter ratio, lowest biomass of woody parts (Figure 3) and, hence, the lowest GE (Figure 6). Sycamore had the highest values of individual leaf mass weight and area as well as total tree foliage area.

In fact, these four tree species represent rather contrasting ecological demands, especially regarding their drought resistance, demands for nutrients and light conditions (e.g., [25,27]). Specifically, common aspen mostly grows under full light conditions but can tolerate partly shaded locations and belongs to pioneer tree species (early successional stands [27]). Similarly, silver birch is a light-demanding pioneer tree species. In our study, birch trees accumulated substantially less woody biomass than aspen trees (Figure 4), which is in accordance with older findings (e.g., [28,29]). Other studies, however, found that aspen grew more slowly than birch [30]. Their production depends on soil conditions. Aspen does not tolerate acid, nutrient-poor, dry or poorly drained soils [28,31,32], while birch was found to grow well also on acidic soils [33]. All stands selected for this study were growing at moderately fertile sites on mesotrophic Cambisols. Hence, we assume that the revealed differences between species did not result from soil conditions. However, a more detailed study on the impact of soil factors, such as soil acidity, on species traits would be required in future. Sycamore typically prefers semi–shaded conditions, but in juvenile stages it can survive also shaded conditions for a long time [34]. It is a fast-growing species [25], which encounters rapid height growth in the early development at ages below 20–25 years [34], which is also documented in our data (Figure 1). European hornbeam is one of the few strongly shade-tolerant native trees in Europe. Interestingly, although it was the most shade-tolerant species among all tree species studied in this work, it manifested the smallest individual leaf area as well as the smallest value of SLA. The smallest SLA values for hornbeam were also found in the multispecies study of Stiegel and Mantilla-Contreraras [26], who concluded that it is the result of the adaptation strategy of this species to survive under warm and dry environmental conditions. Its leaves are more tough [26], and its sap flow is smaller than the flow in sycamore or European beech (*Fagus sylvatica* L.) which makes it less sensitive to drought [35].

Under European conditions, SLA of European beech has been the most studied from among broadleaved species [11,12,36,37]. In contrast, literature search suggested only sparse data about some of four broadleaved species included in our work. Howard [38] showed values for mature trees of silver birch and sycamore of about 250 and 300 cm^2^ g^−1^, respectively. A similar range of SLA values was found for young sycamore by Petritan et al. [39]. While Kinney and Lindroth [40] showed SLA of young aspens of about 170 cm^2^ g^−1^, Stiegel and Mantilla-Contreraras [26] estimated SLA of young hornbeams near 150 cm^2^ g^−1^. Besides other interactions, SLA was proven to be negatively correlated with tree size [41,42] and with tree age [43,44]. However, more research is needed to better understand how leaf properties and biomass allocation are related given different ecological demand of these species.

Our model for GE (Figure 6) indicated much higher values for hornbeam than for other species under the conditions of the same diameter. The differences between hornbeam and other species were about two- and threefold depending on a diameter. Unfortunately, we lack data on real annual diameter increments at an individual tree level, and thus, our models for GE are rather theoretically based under the expectation of the same increment. However, the applied values of diameter increment (5 and 10 mm) should be close to most frequent situations in field conditions. Another limitation of our approach is the assumption that a diameter increment does not change with tree size (quantified by diameter *d*_0_). Hence, the actual reduction in GE of small individuals (with diameters up to approximately 20 mm) with the increasing diameter might be less steep than in our model. Previously, we focused on modelling GE within a European beech stand [41]. There, the size of beech trees characterized by diameter *d*_0_ was mostly related to a tree position in the stand (bio–sociological position). The regression model indicated increasing GE with diameter *d*_0_ that very probably corresponds to light availability [41]. This is in accordance with rather old findings by Waring [45] that showed a negative relationship between leaf area index (LAI; stand leaf area expressed per m^2^ of ground surface) and GE. Since a large value of LAI indicates a denser canopy and more intense overlapping of foliage, less light would be expected along the vertical stand profile.

Works showing details about GE in terms of tree size either in ontogenetic aspects or within stand hierarchy are scarce. Xu et al. [46] showed a decreasing GE in *Quercus*-dominated stands with projected leaf area. Currently, a decreasing GE (expressed as woody biomass produced per leaf mass) with an increasing stand age has been shown in mature stands (50–150 years) of five tree species [47]. Interspecific comparisons of GE in forest trees are still missing, although this kind of results would help to elucidate ecological demands and growth strategies of particular species [18].

Data obtained from empirical studies such as those presented here are also important from the standpoint of modelling forest dynamics. Experiments focusing on multiple species can provide modellers with valuable information and knowledge that can be implemented in models of vegetation dynamics in the form of species-specific parameterisation. Forest growth models are frequently parametrised only for a small number of the most common tree species. Therefore, including less abundant species into models can increase their generality [48] and applicability to simulate forests of different tree species as well as mixed forests [49]. This feature is of particular importance under the ongoing climate change, which will modify environmental conditions of some regions at an unprecedented level [50,51] and may cause substantial changes in tree species composition [52,53].

## 4. Materials and Methods

### 4.1. Sampling and Data Collection

**Figure 7:**
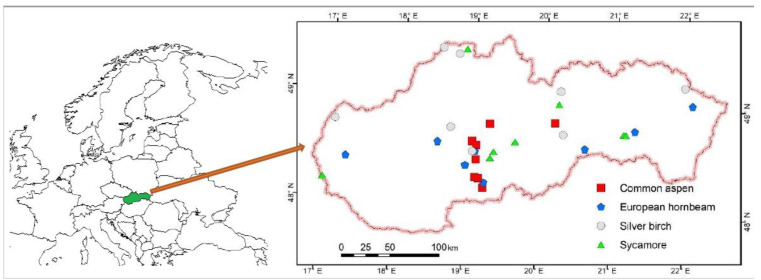
Location of plots used for sampling biomass of four broadleaved tree species in the area of Slovakia.

Four tree species, namely common aspen, European hornbeam, silver birch and sycamore, were included in our study. Firstly, we selected several at least twelve naturally regenerated, even-aged high forest stands for each target tree species that were in initial development stages from the regeneration stage up to the thicket stage (in Slovakia this stage is characterized with a mean stand diameter up to 12 cm). The selection was performed using the current national database of forest stands based on the data from Forest Management Plans (see also http://gis.nlcsk.org/lgis/). At the same time, the stands had to grow at moderately fertile sites; practically all of them grew on mesotrophic Cambisols. In fact, since mesotrophic Cambisols cover about 2/3 of forest area in Slovakia [54], we exclusively selected sites with these soil conditions. Then, we visited and inspected the individual stands and selected eight stands for each species (Figure 7) based on the following criteria: contribution of target species to stand density at least 90%, no or minimum damage by pests—especially by game browsing, stand compactness (i.e., no continuous treeless gaps) and self-development since forest establishment (i.e., the complete absence of past silvicultural measures, e.g., thinning, during the existence of the current stand).

Tree sampling was conducted every year between 2016–2020, always in the second half of the growing season, especially in August and early September. The timing for harvest was chosen to sample trees with fully developed foliage. Each tree species at a specific location was represented by about 20 individuals. Hence, in total we sampled between 150 and 200 trees per species (Table 6). Sample trees were selected in each stand randomly, avoiding damaged, dying, deformed or atypically shaped individuals, e.g., ones growing on stand edges. We included trees of all bio–sociological positions, i.e., dominant, codominant, intermediate and suppressed individuals. We avoided only severely suppressed individuals with early symptoms of dieback.

Each selected tree was cut at the ground level. Its diameter at stem base (diameter *d*_0_) was measured using a digital calliper with an accuracy of 0.1 mm. Branches were cut with garden scissors, and a tree height was measured using a metal tape measure with an accuracy of 1.0 mm. The root system was excavated. Nine leaves were cut off from each sampled tree, three pieces per crown third (upper, middle and lower part of crown). Leaves were collected from most of the sampled trees. The collected leaves were inserted into laboratory envelopes with labels indicating a tree code and position in the tree crown. Afterwards, all tree components were packed in labelled paper bags and transported to our laboratory.

Sampled fresh leaves (i.e., nine pieces from each tree) were scanned, and their area was calculated using the Easy Leaf Area program [55] with an accuracy of 0.1 mm^2^. Then, each leaf was inserted in a separate envelope and oven-dried under temperature of 95 °C for 24 h. Dried leaves were weighed with laboratory scales (accuracy of 0.001 g). All other leaves from each tree were manually picked from branches, packed, dried in the same way as subsamples of leaves and weighed (accuracy of 0.01 g). Sampled woody parts (branches, stems and roots) were dried in a large-capacity drying oven to reach the constant weight (under a temperature of 95 °C for 120 h). Afterwards, they were weighed with an accuracy of 0.1 g.

### 4.2. Data Analyses and Modelling

Model developing focused on biomass quantification of foliage and woody parts of individual tree species and on quantification of leaf traits. Models were derived for two levels:

- A level of individual leaves (for leaf mass weight *w_f_* expressed in g, leaf area *LA* in cm^2^ and *SLA* in cm per g);
- A tree level (leaf mass weight *w_f_* expressed in kg, mass weight of woody parts *w_wp_* in kg, tree mass weight *w_w_* in kg, *LA* in m^2^, *LAR* in m^2^ of leaf area per kg of woody parts, *LMR* in kg of leaves per kg of woody parts).

The relationship between variables was described with an allometric equation:

(1) $$Y=b_{1}X^{b_{2}}$$

where *Y* is a dependent variable of those listed above, *b*_1_ and *b*_2_ are regression coefficients and *X* is a stem base diameter *d*_0_ (expressed in mm) in all models. Most models were derived in the basic power form with the regression analysis by fitting the Equation (1) to respective empirical data using Statistica 10.0 Program (StatSoft, Tulsa, OK, USA). Equations for the calculation of foliage and woody parts biomass were derived in the linearised logarithmic form of the allometric equation as follows:

(2) $$lnY=b_{0}+b_{2}\cdot lnX+\varepsilon$$

where *b*_0_ = *ln* *b*_1_, and ε = *ln* θ is the error (additive error term).

This approach was chosen to ensure methodological consistency with previous works quantifying biomass components of young trees (Pajtík et al. [56]). The advantage of the logarithmic form of Equation (2) is that the parameters can be estimated using a linear regression. In addition, the logarithmic transformation compensates for the tendency to accelerate the increase of the dependent variable with the tree size and, hence, the heteroscedasticity of residuals, which is always present in the case of this data type. Thanks to this approach, the model satisfies the assumption of constant variance. However, the logarithmic transformation of the dependent variable causes bias, which occurs after the inverse transformation of the logarithmic form to the original one. Hence, when Equation (2) is transformed back, it needs to be corrected for the logarithmic bias. For this purpose, a correction factor referred to as λ is used in the retransformation:

(3) $$Y=e^{\left(b_{0}+b_{2}\cdot lnX\right)}\cdot \lambda$$

The correction factor was calculated according to Marklund [57] using Equation (4):

(4) $$\lambda =\frac{\sum_{i=1}^{n}Y_{i}}{\sum_{i=1}^{n}e^{ln{\hat{Y}}_{i}}{}_{}}$$

where *n* is the number of trees. In the case of biomass calculation at a tree level (that is our case in this work), Ledermann and Neumann [58] recommend using Equation (5):

(5) $$\bar{\lambda }=\frac{1}{n}\sum_{i=1}^{n}\frac{Y_{i}}{e^{ln{\hat{y}}_{i}}}$$

Models at an individual leaf level were derived from measured data of *d*_0_, weight *w_f_* and *LA* of all sampled leaves (Table 2) and separately for the top, middle and bottom thirds of the crown (Appendix A, Table A1)*. SLA* was calculated as follows:

(6) $$SLA=\frac{LA}{w_{f}}$$

Since, at a tree level, only diameters *d*_0_ and mass weights *w_f_* were determined, leaf area *LA* of the whole tree was calculated using the following Equation (7):

(7) $$LA=SLA\cdot w_{f}$$

Here, we derived a new model using the measured mass weight of all leaves of a particular tree and the modelled *SLA* (Table 4).

*LMR* is defined as a ratio between the foliage biomass and the total tree biomass:

(8) $$LMR=\frac{w_{f}}{w_{w}}$$

Similarly, *LAR* is a ratio between the tree leaf area and the total tree biomass:

(9) $$LAR=\frac{LA}{w_{w}}$$

The allometric Equation (1) was used to describe the relationship between the diameter *d*_0_ and *LMR* or *LAR*.

The annual production of wood biomass $\Delta w_{wp}$ was calculated as a difference between the biomass of a tree with a diameter *d*_0_ + 5 mm or *d*_0_ + 10 mm ($w_{wp\left(d_{0}+5\right)})$ and the biomass of a tree with a diameter *d*_0_ $\left(w_{wp\left(d_{0}\right)}\right)$, which were calculated using allometric models derived for the biomass of woody parts $w_{wp}$ presented in Table 2, as follows:

(10) $$\Delta w_{wp}=w_{wp\left(d_{0}+5\right)}-w_{wp\left(d_{0}\right)}$$

The values 5 and 10 mm represent hypothetical annual diameter increments that were selected for all species to allow the interspecies comparison based on the average diameter increments that were observed for these species in the initial growing stage. The two values represented a range of mean increments derived from sampled trees by dividing tree diameter with its age.

Growth efficiency *GE* quantifies annual production of wood biomass per leaf area unit:

(11) $$GE=\frac{\Delta w_{wp}}{LA}$$

*GE* was derived using the models of wood biomass (Table 3) and total tree leaf area (Table 4) with regard to diameter *d*_0_.

## 5. Conclusions

Our study suggested differences but also similarities in foliage traits among species. Specifically, while aspen and birch had rather similar foliage properties, sycamore and hornbeam differed from the others. Intraspecific differences were related to tree size and leaf position along the vertical crown profile. Models for GE suggested a steep decrease with the increasing stem diameter as well as considerably greater values for hornbeam than for the rest of species. However, the model assumed the same diameter increment for considered tree sizes and tree species. This situation probably does not correspond to real field conditions. Hence, future GE models should utilize observed values of diameter increment and account for the bio–sociological position of a tree within the stand. Further GE modelling would elucidate the role of the stand structure and tree species mixture for ecological and production properties of forest stand. Although this kind of GE research and related modelling is still in a very early stage of development, our work might be understood as a good start and especially as an example of methodological approaches in this field. The information of GE plays a crucial role in current management challenges linked to temporary deforestation caused by accelerating disturbances due to the ongoing climate change. We believe that the outputs from further modelling would help stakeholders in making decisions about optimal forest management including planning suitable tree species composition for reforestation and silvicultural measures aiming at diversification of stand structure that would increase forest resilience in future and would promote biodiversity and carbon sequestration.

## Figures and Tables

**Table 1:** Basic statistical characteristics of relationships between diameter at stem base and tree height derived for four broadleaved species using equation in Figure 1d. Here, *b*_0_, *b*_1_ and *b*_2_ are regression coefficients; S.E. is standard error; P is *p*-value; R^2^ is coefficient of determination and MSE is mean square error.

Tree Species	*b*_0_	S.E.	P	*b*_1_	S.E.	P	*b*_2_	S.E.	P	R^2^	MSE
Common aspen	8.221	11.812	0.487	7.077	0.693	<0.001	0.021	0.009	0.017	0.897	0.612
European hornbeam	16.924	6.036	0.006	3.254	0.527	<0.001	0.093	0.009	<0.001	0.864	0.485
Silver birch	−11.136	20.249	0.583	10.251	1.214	<0.001	0.046	0.014	<0.001	0.776	0.840
Sycamore	84.237	25.897	0.001	3.273	1.311	0.014	0.073	0.014	<0.001	0.882	0.749

**Table 2:** Species-specific models for estimating individual leaf weight (g), leaf area (cm^2^) and specific leaf area (SLA in cm^2^ g^−1^) based on using equation in Figure 1d using diameter at stem base *d*_0_ (mm) as a predictor. Meaning of abbreviations is explained in Table 1.

Leaf Trait	Tree Species	*b*_0_	S.E.	P	*b*_1_	S.E.	P	R^2^	MSE
Weight (g)	Aspen	0.018	0.003	<0.001	0.552	0.034	<0.001	0.199	0.005
	Hornbeam	0.013	0.001	<0.001	0.606	0.029	<0.001	0.232	0.003
	Birch	0.042	0.004	<0.001	0.184	0.025	<0.001	0.197	0.003
	Sycamore	0.304	0.039	<0.001	0.189	0.035	<0.001	0.029	0.168
Area (cm^2^)	Aspen	4.790	0.495	<0.001	0.390	0.027	<0.001	0.183	59.3
	Hornbeam	1.588	0.091	<0.001	0.342	0.018	<0.001	0.222	3.48
	Birch	8.304	0.651	<0.001	0.099	0.021	<0.001	0.125	41.7
	Sycamore	60.791	5.707	<0.001	0.157	0.026	<0.001	0.037	2921
SLA (cm^2^ g^−1^)	Aspen	457.011	31.852	<0.001	−0.284	0.020	<0.001	0.172	2174
	Hornbeam	98.600	3.904	<0.001	−0.122	0.014	<0.001	0.060	506
	Birch	211.803	5.334	<0.001	−0.084	0.007	<0.001	0.291	943
	Sycamore	205.480	11.766	<0.001	0.004	0.026	0.822	0.000	4992

**Table 3:** Species-specific biomass models for leaves, woody parts (i.e., branches, stem and roots) and a whole tree based on Equation (2) with diameter at stem base *d*_0_ as a predictor. λ is logarithmic transformation bias, and the meaning of other abbreviations is explained in Table 1.

Tree Species	Compartment	*b*_0_	S.E.	P	*b*_2_	S.E.	P	R^2^	MSE	λ	S.D.
Common aspen	Leaves	−2.907	0.225	<0.001	2.020	0.069	<0.001	0.829	0.429	1.191	0.634
	Woody parts	−2.760	0.093	<0.001	2.699	0.028	<0.001	0.982	0.071	1.036	0.293
	Whole tree	−2.379	0.092	<0.001	2.618	0.028	<0.001	0.981	0.070	1.030	0.296
European hornbeam	Leaves	−4.127	0.165	<0.001	2.354	0.061	<0.001	0.884	0.497	1.225	0.700
	Woody parts	−2.604	0.064	<0.001	2.680	0.024	<0.001	0.985	0.072	1.037	0.391
	Whole tree	−2.389	0.062	<0.001	2.640	0.023	<0.001	0.986	0.068	1.034	0.277
Silver birch	Leaves	−2.404	0.154	<0.001	1.904	0.044	<0.001	0.913	0.191	1.089	0.422
	Woody parts	−2.332	0.116	<0.001	2.449	0.033	<0.001	0.970	0.106	1.053	0.345
	Whole tree	−2.715	0.128	<0.001	2.742	0.029	<0.001	0.973	0.122	1.034	0.370
Sycamore	Leaves	−2.468	0.135	<0.001	1.894	0.043	<0.001	0.928	0.247	1.118	0.509
	Woody parts	−3.322	0.081	<0.001	2.816	0.028	<0.001	0.986	0.101	1.052	0.347
	Whole tree	−2.558	0.073	<0.001	2.627	0.023	<0.001	0.988	0.072	1.037	0.293

**Table 4:** Species-specific models for estimating total leaf area at a tree level (m^2^) based on equation in Figure 1d using diameter at stem base *d*_0_ as a predictor. Meaning of abbreviations is explained in Table 1.

Tree Species	*b*_1_	S.E.	P	*b*_2_	S.E.	P	R^2^	MSE
Common aspen	0.0031	0.0012	0.010	1.723	0.093	<0.001	0.734	0.834
European hornbeam	0.0003	0.0001	<0.001	2.060	0.063	<0.001	0.889	0.014
Silver birch	0.0015	0.0006	0.011	1.883	0.088	<0.001	0.821	1.236
Sycamore	0.0018	0.0007	0.011	1.884	0.090	<0.001	0.838	0.807

**Table 5:** Species-specific models for leaf mass ratio (LMR) and leaf area ratio (LAR) based on equation in Figure 1d using diameter at stem base *d*_0_ as a predictor. Meaning of abbreviations is explained in Table 1.

Indicator	Tree Species	*b*_1_	S.E.	P	*b*_2_	S.E.	P	R^2^	MSE
Leaf mass ratio (LMR)	Aspen	0.657	0.083	<0.001	−0.585	0.048	<0.001	0.458	0.0031
	Hornbeam	0.228	0.020	<0.001	−0.319	0.038	<0.001	0.221	0.0030
	Birch	0.591	0.059	<0.001	−0.433	0.034	<0.001	0.477	0.0028
	Sycamore	0.890	0.032	<0.001	−0.623	0.019	<0.001	0.865	0.0026
Leaf area ratio (LAR)	Aspen	308.403	37.187	<0.001	−0.869	0.051	<0.001	0.634	165.93
	Hornbeam	26.542	1.811	<0.001	−0.497	0.034	<0.001	0.444	18.39
	Birch	122.745	11.885	<0.001	−0.512	0.034	<0.001	0.560	75.71
	Sycamore	184.313	6.751	<0.001	−0.618	0.019	<0.001	0.862	112.20

**Table 6:** Main characteristics on sampled locations, sampled trees and scanned leaves for individual investigated tree species.

Tree Species	Range of Altitudes	Number of	Mean Ages * of	Number of	Number of	Mean Value and (Standard Deviation) of	
	(m a.s.l.)	Stands	Stands	Sampled Trees	Scanned Leaves	Tree Height (m)	Diameter *d*_0_ (mm)
Common aspen	335–870	8	2–12	185	980	3.81 (2.42)	31.39 (20.00)
European hornbeam	295–570	8	1–10	200	1392	2.68 (1.83)	17.84 (13.94)
Silver birch	260–950	8	1–12	178	1476	3.12 (1.93)	38.49 (25.69)
Sycamore	415–970	8	2–11	150	1009	3.00 (2.30)	27.73 (22.18)

Note: * Mean age of each stand selected for tree sampling was taken from Forest Management Plans (approximate values).

## Appendix A

**Table A1:** Species-specific allometric models for leaf weight (g), leaf area (cm^2^) and SLA (cm^2^ g^−1^) in three crown parts using diameter at stem base *d*_0_ as a predictor (mm). Meaning of abbreviations is explained in Table 1.

**Common Aspen**									
Leaf Trait	Crown Part	*b*_0_	S.E.	P	*b*_1_	S.E.	P	R^2^	MSE
Weight (g)	Lower	0.007	0.002	<0.001	0.712	0.056	<0.001	0.342	0.002
	Middle	0.015	0.003	<0.001	0.585	0.048	<0.001	0.332	0.003
	Upper	0.036	0.008	<0.001	0.441	0.059	<0.001	0.161	0.008
Area (cm^2^)	Lower	2.611	0.470	<0.001	0.519	0.047	<0.001	0.285	45.98
	Middle	4.113	0.640	<0.001	0.430	0.041	<0.001	0.265	44.10
	Upper	8.869	1.562	<0.001	0.255	0.047	<0.001	0.090	74.35
SLA (cm^2^ g^−1^)	Lower	439.320	45.321	<0.001	−0.233	0.029	<0.001	0.167	2077
	Middle	484.466	51.303	<0.001	−0.297	0.030	<0.001	0.225	1717
	Upper	469.014	52.938	<0.001	−0.342	0.032	<0.001	0.249	1354
**European Hornbeam**									
Leaf trait	Crown part	*b*_0_	S.E.	P	*b*_1_	S.E.	P	R^2^	MSE
Weight (g)	Lower	0.011	0.0015	<0.001	0.471	0.040	<0.001	0.226	0.0006
	Middle	0.012	0.0015	<0.001	0.599	0.038	<0.001	0.346	0.0015
	Upper	0.016	0.0020	<0.001	0.665	0.036	<0.001	0.434	0.0043
Area (cm^2^)	Lower	1.265	0.119	<0.001	0.327	0.029	<0.001	0.222	1.699
	Middle	1.477	0.124	<0.001	0.369	0.026	<0.001	0.307	2.364
	Upper	1.935	0.156	<0.001	0.347	0.025	<0.001	0.314	3.974
SLA (cm^2^ g^−1^)	Lower	98.892	5.507	<0.001	−0.064	0.019	<0.001	0.027	362
	Middle	112.295	7.540	<0.001	−0.159	0.023	<0.001	0.107	397
	Upper	97.470	5.718	<0.001	−0.196	0.021	<0.001	0.161	388
**Silver Birch**									
	Crown part	*b*_0_	S.E.	P	*b*_1_	S.E.	P	R^2^	MSE
Weight (g)	Lower	0.026	0.0035	<0.001	0.180	0.036	<0.001	0.052	0.0007
	Middle	0.042	0.0048	<0.001	0.152	0.031	<0.001	0.051	0.0010
	Upper	0.056	0.0074	<0.001	0.207	0.035	<0.001	0.077	0.0034
Area (cm^2^)	Lower	5.096	0.589	<0.001	0.134	0.031	<0.001	0.039	14.07
	Middle	7.940	0.779	<0.001	0.089	0.027	0.001	0.023	18.75
	Upper	11.878	1.376	<0.001	0.090	0.032	0.005	0.018	58.59
SLA (cm^2^ g^−1^)	Lower	203.850	8.148	<0.001	−0.050	0.011	<0.001	0.038	895
	Middle	207.530	8.800	<0.001	−0.079	0.012	<0.001	0.079	885
	Upper	228.310	8.925	<0.001	−0.132	0.011	<0.001	0.214	668
**Sycamore**									
Leaf trait	Crown part	*b*_0_	S.E.	P	*b*_1_	S.E.	P	R^2^	MSE
Weight (g)	Lower	0.356	0.122	0.004	0.024	0.092	0.796	0.226	0.0002
	Middle	0.296	0.080	<0.001	0.189	0.071	0.008	0.022	0.113
	Upper	0.209	0.031	<0.001	0.366	0.041	<0.001	0.156	0.188
Area (cm^2^)	Lower	61.590	17.276	<0.001	0.087	0.075	0.247	0.005	2241
	Middle	66.233	13.586	<0.001	0.145	0.054	0.008	0.025	2380
	Upper	49.257	5.536	<0.001	0.251	0.032	<0.001	0.121	3092
SLA (cm^2^ g^−1^)	Lower	190.634	28.250	<0.001	0.071	0.039	0.073	0.014	5375
	Middle	207.066	28.783	<0.001	0.0045	0.037	0.904	0.0001	4092
	Upper	286.219	18.400	<0.001	−0.139	0.021	<0.001	0.088	3556
